# Effects of polio eradication activities on routine immunization: lessons from the 2013 outbreak response in Somali region of Ethiopia

**DOI:** 10.11604/pamj.supp.2017.27.2.10569

**Published:** 2017-06-09

**Authors:** Belete Tafesse, Ephrem Tekle, Liya Wondwossen, Mengistu Bogale, Braka Fiona, Peter Nsubuga, Karengera Tomas, Aron Kassahun, Gallagher Kathleen, Aschalew Teka

**Affiliations:** 1 World Health Organization, Ethiopia; 2 Ministry of Health, Ethiopia; 3 Global Public Health Solutions LLC, Decatur GA, USA

**Keywords:** Disease eradication, immunization, outbreak, polio, Ethiopia

## Abstract

**Introduction:**

Ethiopia experienced several WPV importations with a total of 10 WPV1 cases confirmed during the 2013 outbreak alone before it is closed in 2015. We evaluated supplemental immunization activities (SIAs), including lessons learned for their effect on the routine immunization program during the 2013 polio outbreak in Somali regional state.

**Methods:**

We used descriptive study to review documents and analyse routine health information system reports from the polio outbreak affected Somali regional state.

**Results:**

All data and technical reports of the 15 rounds of polio SIAs from June 2013 through June 2015 and routine immunization coverages for DPT-Hib-HepB 3 and measles were observed. More than 93% of the SIAs were having administrative coverage above 95%. The trend of routine immunization for the two antigens, over the five years (2011 through 2015) did not show a consistent pattern against the number of SIAs. Documentations showed qualitative positive impacts of the SIAs strengthening the routine immunization during all courses of the campaigns.

**Conclusion:**

The quantitative impact of polio SIAs on routine immunization remained not so impressive in this study. Clear planning, data consistencies and completeness issues need to be cleared for the impact assessment in quantitative terms, in polio legacy planning as well as for the introduction of injectable polio vaccine through the routine immunization.

## Introduction

The global burden of poliomyelitis has decreased by ≥ 99% since the time the World Health Assembly endorsed the initiative for global polio eradication in 1988. The burden of the wild poliovirus (WPV) has shown significant reduction in Africa with the last case confirmed in July 2014 in Nigeria. Ethiopia joined the polio eradication effort in 1996 and was able to interrupt endemic WPV transmission in 2001. However, the country experienced several WPV importations where between 2004 and 2008 a total of 44 cases were confirmed associated with six different importations. The last importation of WPV was in August 2013 following the outbreak that was declared in the Horn of Africa in April 2013. A total of 10 WPV 1 cases were confirmed with onset of paralysis of the last case on 5 January 2014.

Since the late 1980s, use of Supplemental Immunization Activities (SIAs) has been a key strategy of the Global Polio Eradication Initiative (GPEI). Polio SIAs are mass vaccination campaigns that aim to administer additional doses of Oral Poliovirus Vaccine (OPV) to each child (usually aged <5 years), regardless of their vaccination history. In doing so, SIAs attempt to remedy the limited ability of routine immunization services to reach at-risk children with the number of OPV doses required to generate immunity. Several rounds of polio SIAs were conducted in Ethiopia following each outbreak as per the recommendation of the polio Global Advisory Committee. Like the majority of polio outbreaks which were controlled within 6 months with OPV, it took about five months in the country to limit further new incidence of the WPV; however the outbreak response extended to 2 years between June 2013 and June 2015 and the country responded with 15 SIAs out of which four were National Immunization Days (NIDs) [1]. As in many other countries, the high quality responses were partly due to the appointment of staff to oversee implementation, engagement of the national government and its partners as well as participation of pastoralist community leaders [2]. Deployment of national stop transmission of polio (STOP) team members with rich experience in surveillance and routine immunization helped to sustain the gains of SIAs integrated with routine services [3]. A robust acute flaccid paralysis surveillance system, including a multi-tiered polio laboratory network, has been maintained, forming the platform for integrating measles, neonatal tetanus, and other vaccine-preventable disease surveillance and their respective control goals in the WHO Western Pacific Region [4]. Resources need to be mobilized and invested in surveillance and routine immunization systems parallel with SIAs to avert a risk for additional outbreaks of WPV and other vaccine-preventable illnesses in a region. Monitoring and evaluation of program strengthening activities are needed [5].

Prior use of routine immunization services and compliance with the routine OPV schedule has strong positive association with SIA participation [6]. On the other hand, the few studies conducted elsewhere did not find compelling evidence of widespread and significant effects of polio eradication campaigns, either positive or negative, on quantitative measures of routine immunization coverage and other maternal healthcare [7]. A study conducted on the effects of extra immunization activities on routine immunization coverage rate of the third dose of diphtheria-pertussis-tetanus (DPT3) showed the districts that implemented extra national neonatal tetanus immunization were at risk of having lower routine DPT3 coverage than those that did not [8]. The different studies conducted elsewhere have a mixed outcome of effects of the polio SIA on routine immunization; moreover, such documentations appear to be inexistent or patchy in Ethiopia. Therefore, this study will help increase knowledge of implementers to look for effects of SIAs on routine immunization in Ethiopian context and also helps in planning SIAs making the agenda of positive impact of the campaigns for sustainability and program ownership purposes from the outset. In this article, we set out as an objective to document the effects of the polio SIAs on routine immunization services (both qualitatively and quantitatively) during the 2013 polio outbreak affected regional state of the country, Somali.

## Methods

**Study area:** administratively Ethiopia is divided into nine Regional States and two City Administrations. One of the regional states is Somali region and it comprises nine zones. The region shares porous border with Somalia and Kenya which puts it at increased risk of importation of WPV. The total population of the region was estimated to be 5,446,968 in 2015 and the <5 years of age comprised 10.1% of the total population (National census 2007 projection).

**Study design:** we conducted a descriptive study design using campaign and routine health management information system (HMIS) data from June 2013 to June 2015 to explore lessons from the SIAs and observe trends in the routine immunization coverage in relation with the number of polio SIAs conducted in Somali region of Ethiopia.

**Method of sampling and recruitment:** we purposefully selected Somali region of the country as it was epi-center for the polio outbreak during mid-2013. In addition, all rounds of the polio SIAs included the region in the subsequent campaigns until closure of the outbreak in June 2015. Except during the four NIDs conducted from 2013 through 2015, the remaining regions had intermittent polio response campaigns during the 2 years outbreak period.

**Procedures:** we obtained technical reports of all the SIAs conducted as part of the outbreak response in Somali region from June 2013 to June 2015 as well as quarterly program review documents. The technical reports of each of the polio SIAs rounds were reviewed for coverage of the SIAs, lessons learned and best practices out of the campaigns as to their contribution to strengthen the routine immunization were narrated and summarized in tables and graphs. We also compiled the periodic HMIS reports from the region to see the trends in uptake of the routine immunization coverage on key indicators of (i.e., DPT-Hib-HepB3, measles and dropout rates) over the same 2 years period.

**Statistical analyses:** after the data compilation was completed, we checked the data manually for completeness and consistency. We entered the data into an MS Excel spreadsheet and checked for major outliers, and then analysed for proportions, percentages and trends. We used frequency distribution and percentages used to describe the variables, and compiled results and presented those using tables, graphs, and narrations.

## Results

### Summary of the polio SIAs

As shown in Table 1, a total of 15 rounds of polio NIDs were conducted in Somali region between June 2013 and June 2015. On average, the administrative campaign coverage over the 2 years of outbreak response was 97.8% and in all of the campaign rounds, coverage was above the cut off for high coverage (which is 95%) except in July 2013 where it was 92%. On average, among children who got vaccinated in the age range of 0-11 months, 1 child in every 20 had not received any OPV vaccination prior to the campaign, that is they were labelled as zero dose children. The zero dose rate in the age range of 12-59 was 1.6%. This rate was fluctuating in both age groups but higher during the initial rounds of the response (i.e., during June and July 2013).

**Table 1:** Administrative data of polio supplemental immunization activities, Somali region, Ethiopia, June 2013 to June 2015

Rounds of Polio SIAs	OPV coverage %	(N)	Zero dose (%) in 0-11 mo	(N)	Zero dose (%) in 12-59 months	(N)
Jun-13	95.2	883,365	14.8	26,189	5.3	35,336
Jul-13	92.0	818, 582	15.6	24537	5.2	33,767
Aug-13	95.5	805,382	3.2	5,354	5.7	3,586
Oct-13	97.1	822,662	7.3	12,472	4.2	27,618
*Dec-13	98.2	2,376,804	4.2	7,888	0.8	5,324
*Jan-14	96.5	2,476,024	2.5	4,976	0.6	4,451
*Mar-14	99.8	2,638,848	3.0	6,331	0.3	1,886
*May-14	95.5	2,571,328	2.3	4,491	0.2	1,459
Jun-14	99.3	913,676	7.1	4,492	0.5	3,403
Jul-14	97.2	894,169	2.5	4,410	0.2	1,705
Sep-14	100.5	953,892	3.4	6,721	0.4	3,000
Nov-14	98.5	936,416	1.6	5,331	0.1	1,031
Feb-15	98.9	939,461	0.4	5,331	0.1	1,014
Mar-15	103.8	969,498	2.0	6,177	0.1	659
Jun-15	99.7	970,403	3.6	6,736	0.2	1,619
**Average**	**97.8**		**4.9**		**1.6**	

* Under 15 years campaigns

### Integrated routine immunization service delivered during the polio SIA rounds

Routine immunization service provisions were integrated with the SIAs starting from the May 2014 round with all antigens. During their house to house visits, vaccinators sought for unvaccinated or under vaccinated children based on the national schedule for all the routine immunization antigens and referred them to nearby temporary fixed stations established for vaccination. As shown in Table 1, although no targets were set for the routine immunization service tracing during the campaigns, from May 2014 to June 2015, a total of 13,284 and 15,894 children were vaccinated for DPT-Hib-HepB 3 and measles respectively. The routine immunization coverage for the antigens varied in each round, and was highest in May 2014 round and lowest in March 2015 round (Table 2).

**Table 2:** Number of children vaccinated for ri with selected indicators during the sia rounds, Somali region, Ethiopia, June 2013 to June 2015

Rounds of Polio SIAs	DPT-Hib-HepB 3 (N)	Measles (N)
May-14	6,304	8,089
Jun-14	1,184	1,512
Jul-14	866	1,071
Sep-14	2,179	2,398
Nov-14	1,803	1,880
Mar-15	372	374
Jun-15	576	570
**Total**	**13,284**	**15,894**

### Trends in the routine immunization coverage

**Figure 1:**
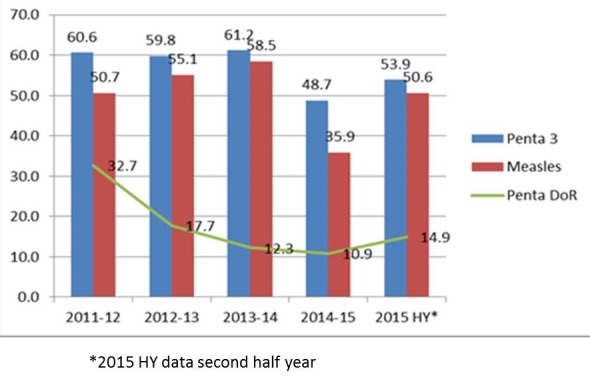
Trend in routine immunization coverage in Somali region, Ethiopia, June 303 2011 to December 2015

The trend of routine immunization for DPT-Hib-HepB3 and measles coverage, over the five years (2011 through 2015) did not show a consistent pattern; however the trend in coverage based on the two indicators was parallel to each other (Figure 1). Throughout the 5 years report periods (2011-2015), the coverages were below the national expectation of 90% as set for DPT-Hib-HepB 3 in 2014. The dropout rates (DoR) of DPT-Hib-HepB1 (Penta 1)-DPT-Hib-HepB3 (Penta 3) were also beyond acceptable set target of 10% in all the report years. However, the DoR for Penta has shown remarkable decline in the years with levelling in later years.

### Report review for lessons learned on how the SIAs supported the routine

***immunization:*** review of the technical reports of the 15 SIA rounds and minutes of the quarterly joint program review meetings of Expanded Program on Immunization (EPI) and surveillance for contributions of the campaigns to support the routine immunization services are stated below.

***Pre-implementation:*** the planning exercises helped to develop or update the social maps in the areas of the campaign. Intense participation of the community at the lower level during microplan development increased the community engagement and program sustainability. Bottom-up microplans were developed or updated in each of the campaigns which were claimed to have boosted the local capacity. During each of the rounds, basic and practical trainings to vaccinators was given where routine immunization and surveillance components were included. All campaign preparation trainings included the following major topics: recording and reporting techniques, vaccine management and distribution, identification of hard to reach and high risk populations, inter personal communication, monitoring and supervision which all boost the routine immunization in general. Deployment of technical assistants with skills in routine immunization and campaigns also helped in coaching and mentoring to the health workers. In majority of the reports of the SIA rounds, immunization task force committees were established up to district level with technical sub working groups. The launching ceremonies for the SIAs and advocacy visits to different level political and community leaders, nongovernmental organizations and other potential partners have helped to gain support and political commitment for immunization as a whole. On the campaign mobilization events, key immunization messages were passed. Functionality and capacity of refrigerators, inventory of cold boxes and vaccine carriers, temperature recording, pattern and placement of routine EPI vaccines were assessed at all levels. In some rural health facilities where electricity supply was not available, kerosene was distributed to start up the refrigerators and maintain the cold chain. In most places, ice pack production was initiated days before the actual implementation of the campaign date. Additionally, in places where nearby health facilities were not available, identification of vaccine distribution points were carried out. In areas where shortage of vaccine carriers was noticed, re-distribution of supplies was made from some health facilities.

***Intra-campaign:*** inter sectoral collaboration among different sectors and partners with mix of technical capacities were increased. School involvement in the campaign helped the partnership by motivating teachers and school administrators for vaccine preventable diseases (VPD). Close supervision and monitoring of the campaigns were also done at all levels which supported monitoring of the routine immunization as well.

***Post campaign:*** in order to evaluate quality of SIAs and take immediate action in low performing areas rapid convenience surveys were conducted where the number of children with zero dozes were monitored and shared for subsequent inclusion in the routine immunization programme. At the end of each SIAs round, regional review meetings were organized with participation of all level supervisors. Strengths and weaknesses were discussed and action points taken for the subsequent round. The routine immunization strengthening efforts were discussed during the review periods.

## Discussion

We found that the administrative coverages in almost all (93.3%) of the 15 polio SIA rounds in Somali region after WPV importations met the cut off for a high quality campaign as supported by the Rapid convenience survey findings as well. Despite this fact the number of zero dose children in both age groups of 0-11 months and 12-59 months of age did not show satisfactory decrease in number along the course of subsequent campaigns. We also found from the campaign coverage trends that lessons and strategies from previous rounds of campaigns were not well documented or not implemented in subsequent campaigns which would have be reflected in an increase of campaign quality/coverage.

We also found that good number of children have got access to routine immunization services integrated with the SIAs as service providers visit the community and link eligible children to prearranged temporary fixed vaccination posts; however the proportion of children accessed and tracked back to the immunization service could not be measured as there were no targets set for vaccination with routine vaccines before the vaccinators went for the activities.

Despite the reported positive qualitative impacts of the campaigns on the political commitment and health workers' knowledge and skill on the routine immunization, the impact of the repeated number of polio SIAs on routine immunization coverages in the region could not be conclusively determined in this study as there was no clear pattern with immunization coverage for the key indicators of DPT-Hib-HepB 3 and measles vaccines. This finding is consistent with the study conducted in seven countries of South Asia and sub-Saharan Africa that assessed impacts of polio eradication activities on key health system functions including routine immunization using mixed methods data.

Despite the major financial and technical investments on immunization in the region, the fact that the study area was already disadvantaged in terms of infrastructures and other facilities due to scattered geographic settlement, pastoralist nature, social insecurity and others could have masked the positive effects of the SIAs on routine immunization in the region with high chance of persistently missing immunization [9, 10]. In addition to the fact that some of the impact of polio campaigns on RI may not immediately translate into immediate improvement in routine immunization coverage, data incompleteness or lack of periodic verification for consistency was also another concern as limitation in this study.

## Conclusion

In conclusion, SIA response should have been more focused in system strengthening and polio eradication activities can provide support for the routine immunization as part of the primary health care; their quantitative impact on routine immunization remained not so impressive in this study. As it is seen in other studies, absence of periodic documentation and tracking effects of the polio SIAs on RI and health system might have contributed to conceal the anticipated positive impacts of the campaigns on the routine immunization following increased commitment to scaling up best practices could lead to significant positive impacts to the routine system [7]. Additional immunization efforts, without additional resources and planned program integration, may not have clear effect on the routine immunization. Even though one of the objectives of the polio SIAs is to strengthen routine immunization, their direct effects were not quantitatively evaluated immediately after each of the campaigns for further action. Having successfully interrupted the indigenous transmission and importation of WPV through SIAs, missing the opportunity to sustain the routine immunization coverage will leave a threat of polio outbreak importations from the remaining polio-endemic countries [11]. Poor vaccination team performance could be one of the major reasons for missing the opportunity to identify the immunization gap in the routine form and link with immunization service points [12]. We recommend that the impact of the polio SIAs on the routine immunization needs to be strongly sought for especially in the process of articulating the lessons learned from the polio eradication efforts during the polio legacy planning. Verification of SIAs data using different mechanisms in pastoralist areas like low cost, hand-held global positioning system (GPS) receivers will increase the reliance of the reported data as was done elsewhere [13]. Data consistencies and completeness issues need to be cleared for the impact assessment in quantitative terms as well as in planning for the introduction of injectable polio vaccine which is to be administered through the routine immunization. Inter-sectoral collaboration between human and animal health services as flexibility and capacity of vaccinators to vaccinate with other routine immunization services when and where nomads were available is commendable [2].

### What is known about this topic

- Use of Supplemental Immunization Activities (SIAs) and strengthening of routine immunization have both been key strategies of the Polio Eradication Initiative;
- Several rounds of polio SIAs were conducted in Ethiopia following each outbreak as per the recommendation with additional intention to strengthen the routine immunization by identifying never-vaccinated children and linking to further completion of their immunization with other infant antigens as well;
- The different studies conducted elsewhere have a mixed outcome of effects of the polio SIAs on routine immunization.

### What this study adds

- This study tries to summarize the qualitative impact of the rounds of polio SIAs in the outbreak focus, Somali region, during the 2013 outbreak period through review of different campaign technical reports and review meeting proceedings;
- Documentation on quantitative impact of polio SIAs appear to be inexistent or patchy in Ethiopia; this study tries to assess impact of the polio SIAs on coverages with regard to DTP3 and measles coverages in routine immunization;
- This study also triggers further statistical analysis to seek associations and statistical significant relations between the polio SIAs and on the routine immunization uptake, the dependent variable.

## Competing interests

Authors declared they have no competing interests. The views expressed in the perspective articles are those of the authors alone and do not necessarily represent the views, decisions or policies of the institutions with which they are affiliated and the position of World Health Organization.
